# Developing diagnostic reference levels in Japan

**DOI:** 10.1007/s11604-020-01066-5

**Published:** 2020-11-19

**Authors:** Reiko Kanda, Masaaki Akahane, Yusuke Koba, Weishan Chang, Keiichi Akahane, Yasuo Okuda, Makoto Hosono

**Affiliations:** 1 National Institute of Radiological Sciences, National Institutes for Quantum and Radiological Science and Technology, 4-9-1, Anagawa, Inage-ku, Chiba, 263-8555 Japan; 2 Department of Radiology, School of Medicine, International University of Health and Welfare, 4-3, Kozunomori, Narita, Chiba 286-8686 Japan; 3 Department of Radiology, Kindai University, 377-2, Ohnohigashi, Osakasayama, Osaka, 589-8511 Japan; 4 Japan Network for Research and Information On Medical Exposures (J-RIME), 4-9-1, Anagawa, Inage-ku, Chiba, 263-8555 Japan

## Introduction

In recent years, concern regarding the effects of radiation exposure in medical care has increased with the rapidly expanding use of medical radiation. Japan is facing a unique situation: although the use of radiological tests is increasing rapidly, there is a great deal of social concern about the potential effects of radiation on human health due to the experiences of exposure to atomic bombs and nuclear disasters.

In the late 2000s, international programs (i.e., the World Health Organization’s Global Initiative and the International Atomic Energy Agency’s (IAEA’s) Smart Card/SmartRadTrack Project) started work on medical radiation levels. It is necessary for Japan as a whole to discuss safety measures with regards to medical exposure. Since there was no legislation mandating the optimization of medical exposure, the Japanese government had not been involved in the promotion of protection against medical exposure. This situation served as an opportunity for establishing a new organization to work on these problems in cooperation with many related communities.

The Japan Network for Research and Information on Medical Exposures (J-RIME) was formed in 2010 to engage the stakeholders, to share information on medical radiation exposure within and outside Japan, and to work towards a national framework for radiation protection from medical exposure.

The J-RIME has established the first DRLs in Japan in June 2015 and published the updated DRLs in July 2020. In this review, the contents of Japan DRLs and their process of the establishment were overviewed.

## The Japan Network for Research and Information on Medical Exposures (J-RIME)

As of 2020, J-RIME has been functioning as a nationwide network with participation from academic institutions, professional societies, national and international organizations and agencies, equipment suppliers, government authorities, individual experts, and other stakeholders. Liaison organizations of J-RIME are increasing year by year (Table 1). So far, the chair of J-RIME has been a member of Committee 3 of the International Commission on Radiological Protection (ICRP), i.e., Dr. Yoshiharu Yonekura (2010–2017) and Dr. Makoto Hosono (2017–present).

**Table 1:** Liaison organizations of J-RIME as of July 2020

Japan Association on Radiological Protection in Medicine	Japanese Society of Nuclear Medicine
Japan Health Physics Society	Japanese Society of Pediatric Radiology
Japan Medical Imaging and Radiological Systems Industries Association	Japanese Society of Radiological Technology
Japan Pediatric Cardiac CT Alliance	The Japan Association of Radiological Technologists
Japan Radiological Society	The Japan Central Organization on Quality Assurance of Breast Cancer Screening
Japan Society of Medical Physics	The Japanese College of Medical Physics
Japanese Society for Oral and Maxillofacial Radiology	The Japanese Radiation Research Society
Japanese Society for Radiation Oncology	The Japanese Society for Neuroendovascular Therapy
Japanese Society of Interventional Radiology	The Japanese Society of Nuclear Medicine Technology

One of J-RIME's activities is to collect medical exposure data arising from radiological procedures in Japanese facilities and to construct a Japanese framework for appropriate protection from medical exposure based on international trends. There are five working groups (WGs): the WG for radiation protection of children to develop diagnostic reference levels (DRLs) for children, the Smart Card WG to examine the Smart Card system to track patient exposure history for Japan, the WG for national survey to survey the actual situations of medical exposure, the WG for the public relations of J-RIME, and the WG for DRL to develop DRLs cooperatively for Japan as a whole. Recently, J-RIME also cooperated with the United Nations Scientific Committee on the Effects of Atomic Radiation (UNSCEAR) Global Survey on Medical Exposure, launched in 2014, which sought to collect all available national information concerning annual numbers of procedures and measures of typical exposure.

## Establishment of Japan DRLs 2015

The establishment of DRLs is an international requirement for protection from medical radiation. Also, in Japan, some medical professionals fully understand the importance of implementing these protective measures. Various societies, researchers, and organizations have performed radiation dose surveys associated with imaging examinations and have independently proposed values for reference dose levels [1–3]. However, none of them have been widely recognized or introduced as national DRLs in Japan.

As described above, it was difficult for protection from medical exposure to be promoted by administrative initiatives or to be authorized officially by government. To establish national DRLs in Japan, therefore, the approval of many related organizations, the professional expertise and collaborating work of many experts including physicians, radiological technologists, and medical physicists, and international certification were necessary.

**Fig. 1:**
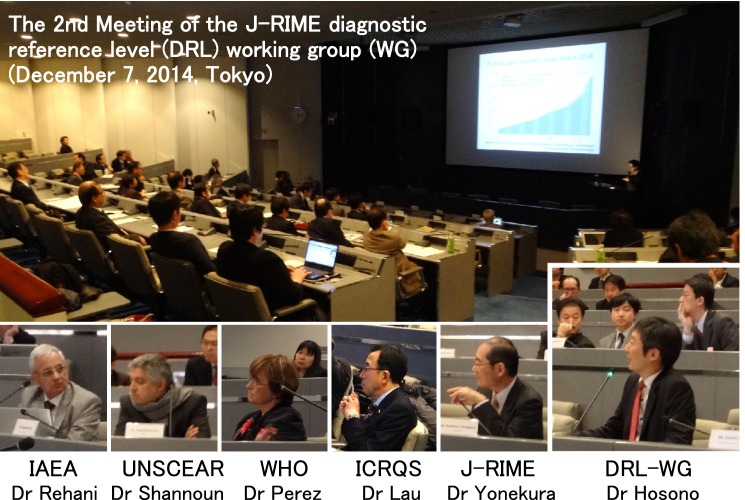
In December 2014, the second meeting of DRL-WG of J-RIME was held and the first national DRLs were discussed with many experts including physicians, radiological technologists, and medical physicists in the form of an open meeting. Experts belonging to international bodies were invited to provide their advice on the process on developing DRLs

Based on the results of the latest nationwide surveys conducted by liaison organizations of the J-RIME, the DRL-WG of J-RIME proposed values of DRLs for computed tomography (CT), general radiography, mammography, dental intraoral radiography, fluoroscopically guided interventional procedures, and nuclear medicine at an open meeting to which experts belonging to international bodies were invited to obtain their advice (Fig. 1).

The set of DRLs approved for publication by the J-RIME and its liaison organizations in June 2015 is called Japan DRLs 2015 (Table 2) [4].

**Table 2:** Comparison of Japan DRLs 2015 and Japan DRLs 2020

		Japan DRLs 2015 [4]	Japan DRLs 2020 [5]
Publication		June 2015	July 2020
Approval		11 organizations	18 organizations
Modality		6	7
Protocol/Scan site/Conditions	CT	6 for adult, 3 for child	8 for adult, 3 for child
	General radiography	6 for adult, 3 for child	11 for adult, 2 for child
	Mammography	1	3
	Dental radiography		
	Intraoral radiography	8 for adult, 8 for 10-year-old child	8 for adult, 8 for 10-year-old child
	Panoramic radiography	–	1
	Dental cone beam CT	–	3
	Interventional radiology	1	
	Head/neck	–	18
	Cardiac regions	–	5 for adult, 2 for child
	Chest and abdomen	–	3
	Diagnostic fluoroscopy	–	12
	Nuclear medicine		
	SPECT	50	51
	PET	13	24
	SPECT/CT	–	9
	PET/CT	–	4
Quantity	CT	CTDI_vol_, DLP	CTDI_vol_, DLP
	General radiography	K_a,e_	K_a,e_
	Mammography	D_G_	D_G_
	Dental radiography		
	Intraoral radiography	K_a,i_	K_a,i_
	Panoramic radiography	–	P_KA_, dose–width product (DWP)
	Dental cone beam CT	–	P_KA_, Air kerma at the iso-center
	Interventional radiology	Rate of K_a,e_ at the interventional reference point (IRP)	K_a,r_, P_KA_, Rate of K_a,e_ at IRP
	Diagnostic fluoroscopy	–	K_a,r_, P_KA_, Fluoroscopy time, Number of images
	Nuclear medicine	Administered dose	Administered dose
	Hybrid CT	–	CTDI_vol_, DLP

*CTDI*_*vol*_ computed tomography dose index (volume), *DLP* dose–length product, *K*_*a,e*_ entrance-surface air kerma, *P*_*KA*_ air kerma-area product, *D*_*G*_ mean glandular dose, *K*_*a,i*_ incident air kerma, *K*_*a,r*_ air kerma at the patient entrance reference point^6^

The J-RIME has promoted better understanding, expanded use, and deeper permeation of DRLs in medical settings. As a result of these activities, J-RIME became widely recognized by the government and the academic community of radiation medicine so that liaison organization increased. Then, J-RIME started to discuss the expansion of target modalities, protocol procedures, and DRL quantities.

## Validation of Japan DRLs 2015

Most of Japan DRLs 2015 for adults were not higher than the national DRLs of other countries and recommendations by European Commission (EC) and the IAEA, except for some CT examinations and myocardial perfusion using ^201^Tl-chloride. However, the reference Japanese man weighs 10–20 kg less than the reference European man, which should be considered for the comparison of values of DRLs among various countries. On the other hand, DRLs for pediatric CT tended to be higher in Japan (Table 3).

**Table 3:** International comparison of DRLs for pediatric CT

	Head			Chest			Abdomen		
	< 1 year	1–5 year	6–10 year	< 1 year	1–5 year	6–10 year	< 1 year	1–5 year	6–10 year
CTDIvol,16 (mGy)									
Japan (2015) [4]	38	47	60	11	14	15	11	16	17
IAEA (2012) [7]	29	37.7	46.1	14.0	16.4*	20.0*	21.4*	26.0*	24.0*
Germany (2006) [8]	33	40	50	3.5	5.5	8.5	5	8	13
Japan (2020) [5]	30	40	55	6	8	13	10	12	15
DLP_16_ (mGy cm)									
Japan (2015) [4]	500	660	850	210	300	410	220	400	530
Germany (2006) [8]	390	520	710	55	110	210	145	255	475
Thailand (2012) [9]	400	570	610	80	140	305	220	275	560
Japan (2020) [5]	480	660	850	140	190	350	220	380	530

^*^The CTD_Ivol,32_ data in the literature is doubled to obtain CTDI_vol,16_

In a survey on DRL comprehension levels six months after the release of Japan DRLs 2015, about 60% of the respondents answered that they knew about the release and 30% understood how to implement Japan DRLs 2015 [10]. In a similar survey two years after the release, about 80% of respondents answered that they knew Japan DRLs 2015, and 40–70% of them had investigated the doses used for CT at their facilities after the DRLs had been established [11]. Many surveys on change in the values of quantities for DRLs were conducted to examine the effect of Japan DRLs 2015. Some surveys conducted after 2017 showed dose reduction, suggesting the contribution of the DRLs 2015 [12]. Considering the review of these follow-up data by the DRL-WG, J-RIME decided at the general meeting of J-RIME in 2018 to update Japan DRLs in 2020.

## Changes in the domestic situation

In Japan, the medical exposure of carers and comforters of patients is stipulated by criteria for patient release based on dose constraints. However, the appropriate management of the medical exposure of patients themselves was not clearly prescribed in law. In April 2017, the Ministry of Health, Labour and Welfare established the “Investigative Committee on Appropriate Management of Medical Radiation,” and conducted discussions until March 2019 on how medical exposure should be appropriately managed. As a result of the committee’s discussions, the following three policies regarding the appropriate management of medical exposure were announced.

1. Regarding the justification and optimization of medical exposure, Government shall clearly stipulate the ensuring of a system for safe management of medical radiation in relevant legislation, such as the Medical Care Act.
2. Specifically, Government shall stipulate training for medical staff involved in radiological diagnosis or treatment, and dose management using DRLs and recording of actual doses related to medical exposure regarding radiological diagnosis or interventional radiology that involves particularly high medical exposure doses.
3. To avoid misinterpretation leading to the restriction of appropriate radiological diagnosis or treatment, information on the justification and optimization of medical exposure should be provided to medical professionals.

An amended ministerial ordinance reflecting the results of the committee’s discussions was published in March 2019, and dose management/dose recording became compulsory with regard to some radiological diagnoses from April 2020. Initially, the committee has discussed policies that the dose recording of patient exposure was made compulsory for all modalities. However, various opinions emerged, such as the burden in clinical settings being high and there being little benefit in recording data in examinations that involve extremely low exposure doses (e.g., dental examinations). Therefore, given that high-dose examinations should be carefully managed, the targets for compulsory dose recording/management were limited to CT scans, fluoroscopic X-ray for angiography, and nuclear medicine examinations.

It was also decided by Government that guidelines formulated by relevant academic societies would be referred to regarding dose management and recording. From now on, in clinical settings, dose management is to be carried out using DRLs established by J-RIME, and quantities used as DRLs are to be recorded. Therefore, J-RIME came to bear more responsibility towards clinical settings than ever before.

## Establishment of Japan DRLs 2020

Publication 135 of the ICRP, which defines the use of DRL in radiological diagnosis, recommends DRL revisions at least every 3–5 years [6]. This is necessary to drive broader optimization by implementing DRLs and to respond to changes in technical progress and clinical demands. In 2018, the J-RIME decided to set 2020, which is five years from the initial version, as the time of revision. Since April 2020, the safety management of medical exposure has been enforced due to the partial revision of the Enforcement Regulations of the Medical Care Law. Thus, Japan DRLs 2020 have been developed in a timely manner with the cooperation of relevant academic societies in a similar manner as that of the DRLs 2015. The features of DRL 2020 compared to DRLs 2015 are described below.

1. The use of ICRP Publication 135 as a reference

In 2017, the ICRP released Publication 135, which comprehensively discusses DRLs [6]. This document provides historical information on the 20 years after the first introduction of the term diagnostic reference level by the ICRP. During the development of the current 2020 DRLs, Publication 135 was used as a reference. For instance, DRLs for pediatric CT were determined not only based on age but also on patient body size.

2. Addition of modality and procedure protocol (Table 2)

DRLs for diagnostic fluoroscopy were newly published in 2020. A nationwide questionnaire on dose, using about 40 protocols of diagnostic fluoroscopy, was conducted, and 12 protocols were selected to set DRLs due to higher patient dose or more patients received. The quantities for use as DRLs for interventional radiology were changed to the quantities the ICRP recommended and DRLs for 28 protocol procedures were determined.

DRLs for panoramic X-rays and cone beam CT in dental radiography and for CT component of hybrid CT (SPECT/CT, PET/CT) were newly established.

In the fields of CT, general radiography, and mammography, the protocol procedures for setting DRLs increased.

## Validation of Japan DRLs 2020

Most of the values of DRLs 2020 were lower than those of DRLs 2015. The main features of DRLs 2020 for each modality are summarized below.

1. CT

The DRLs 2020 for adult CT were reduced compared with DRLs 2015, except DRLs for liver dynamic, which may be due to the increase in the upper weight limit of the reference man (Table 4). Two protocols were selected for new setting of DRLs, i.e., “acute pulmonary thromboembolism and deep vein thrombosis” and “whole body CT for trauma”. These trials were related to the concept of clinical DRL proposed by Eurosafe [13]. This concept is that the DRL is established by clinical purpose rather than organ and may become a major concept in the future.

**Table 4:** The DRLs for adult CT (standard body weight is 50–70 kg)

Protocol	DRLs 2015 [4]		DRLs 2020 [5]	
	CTDIvol [mGy]	DLP [mGy・cm]	CTDI_vol_ [mGy]	DLP [mGy・cm]
General CT				
Routine brain	85	1350	77	1350
Routine chest	15	550	13	510
Chest to pelvis	18	1300	16	1200
Abdomen and pelvis	20	1000	18	880
Liver, multi-phase	15	1800	17	2100
Coronary CTA	90	1400	66	1300
Acute pulmonary thromboembolism and deep vein thrombosis	n/a	n/a	14	2600
Whole body CT for trauma	n/a	n/a	n/a	5800
SPECT/CT (attenuation correction only)				
Brain	n/a	n/a	13.0	330
Heart	n/a	n/a	4.1	85
SPECT/CT (Attenuation correction and image fusion)				
Whole body	n/a	n/a	5.0	380
Brain	n/a	n/a	23.0	410
Head and neck	n/a	n/a	5.8	210
Chest	n/a	n/a	4.1	170
Heart	n/a	n/a	4.5	180
Abdomen, pelvis	n/a	n/a	5.0	210
Extremities	n/a	n/a	4.6	230
PET/CT (Attenuation correction and image fusion)				
Whole body (medical examination)	n/a	n/a	6.1	600
Whole body (medical checkup)	n/a	n/a	5.5	550
Brain (medical examination)	n/a	n/a	31.0	640
Heart (medical examination)	n/a	n/a	9.1	380

The DRLs 2020 for pediatric CT became slightly lower compared with DRLs 2015 (Table 3). The main reason for this reduction may be not optimization using Japan DRLs 2015 but the drastic replacement with CT systems equipped with iterative image reconstruction during the last five years. Compared to the national DRLs of other countries, however, the DRLs 2020 for pediatric CT were still higher.

2. General radiography

Three surveys were conducted for establishing DRLs, with the targets being the training facilities of the Japan Radiological Society, hospitals for occupational health and safety, and those of many other facilities, including clinics [14]. The highest values among three surveys were referred to determine the values of DRLs.

The 75th percentile values of K_a,e_ of the survey on the training facilities of the Japan Radiological Society were 10–40% lower than other two, suggesting there is room for further optimization.

3. Mammography

In Japan, 64% of mammography equipment was used in the facilities certified by the Japan Central Organization on Quality Assurance of Breast Cancer Screening, and dose optimization of mammography is achieved at a considerable level. Therefore, the DRL value was set at the 95th percentile of the distribution of the medians of distributions of mean glandular dose (D_G_) at 40 mm polymethylmethacrylate (PMMA) in 2020 as well as 2015. The value of DRL 2020 for mammography is the same as DRL 2015. The ratio of flat panel detector (FPD) among target equipment of the survey for DRLs 2020 was 51%. The replacement from computed radiography (CR) to FPD may decrease the medians of distributions of D_G_.

DRLs for 2D mammography and digital breast tomosynthesis were newly established using Digital Imaging and Communication in Medicine (DICOM) header file information. Both DRLs were lower than values at 40 mm PMMA. It may be because the ratio of FPD among the target equipment of the survey was 100%.

4. Dental radiology

The DRLs for intraoral X-rays were reduced by 0–20% compared to 2015 values, and the difference in facilities also decreased. A 4- to 14-fold difference in the dose used between the facilities were observed in the survey conducted in 2014 for DRLs 2015. It decreased to 3- to sixfold in the survey conducted in 2018 for DRLs 2020.

The DRLs for panoramic X-ray and dental cone beam CT were newly established and found to be considerably higher than the British DRLs^1^ [15, 16].

5. Interventional radiology

K_a,r_ and P_KA_ have been added to DRLs quantity for interventional radiology in 2020, which are easily available from the imaging equipment, such as parameters displayed on the operator’s console. This change is important for clinical settings because DRL has just been de facto incorporated into Japanese law. Moreover, it enables the comparison of national DRLs among countries (Table 5).

**Table 5:** International comparison of DRLs for interventional radiology

		Japan [5]	France [17]	USA [18]	Germany [19]	Ireland [20]
Diagnostic cerebral angiography	K_a, r_ (mGy)	590	630			
	P_KA_ (Gy・cm2)	89	90			
Embolization in the head for aneurysm	K_a, r_ (mGy)	3100	2770	4750		
	P_KA_ (Gy・cm^2^)	210	190	360	250	62
Diagnostic coronary angiography	K_a, r_ (mGy)	700		1000		
	P_KA_ (Gy・cm^2^)	59		49.4	28	55
Percutaneous coronary intervention (PCI)	K_a, r_ (mGy)	1800		3000		
	P_KA_ (Gy・cm^2^)	130		98.4	48	75
PCI – chronic total occlusion (CTO)	K_a, r_ (mGy)	3900				
	P_KA_ (Gy・cm^2^)	280				350
Hepatic chemoembolization (transcatheter arterial chemoembolization; TACE)	K_a, r_ (mGy)	1400	990	1900		
	P_KA_ (Gy・cm^2^)	270	250	400	230	300
Thoracic endovascular aortic Repair (TEVAR)	K_a, r_ (mGy)	830				
	P_KA_ (Gy・cm ^2^)	200			230	
Endovascular aortic repair (EVAR)	Ka, r (mGy)	1000				
	P_KA_ (Gy・cm ^2^)	210			230	159

The K_a,r_ and P_KA_ of postoperative diagnostic angiography in the head/neck region were 15–40% lower than those of the preoperative one. It may be because the postoperative procedure is performed by narrowing the irradiation field to the target site. Therefore, the DRLs were set separately for the pre- and postoperative procedures.

DRLs for cardiac regions in pediatric patients were newly established according to age and clinical purposes (diagnostic catheterization or interventional radiology).

6. Diagnostic fluoroscopy

About 40% of the fluoroscopes currently operating in Japan can display K_a,r_ and P_KA_ so that the fluoroscopy time and number of images were also used as a DRL quantity.

The fluoroscopy time for endoscopic retrograde cholangiopancreatography (ERCP; diagnosis) were about six times higher than the proposed value of ICRP Publication 117 [21].

The comparison of Japan DRLs for barium enema and ERCP (diagnosis and treatment) with other countries such as Austria, Switzerland, Bulgaria, Cyprus, the Czech Republic, Germany, Denmark, Lithuania, Norway, Sweden, and the United Kingdom revealed that Japan DRLs is within the range of the national DRLs of these countries [22].

7. Nuclear medicine

The DRLs for ^99m^Tc of thyroid examination, ^201^Tl of examination of myocardial blood flow and tumors, and ^67^ Ga of test of tumors/inflammation were significantly reduced compared to Japan DRLs 2015, suggesting the optimization using Japan DRLs 2015.

Hybrid CT DRLs were determined separately for attenuation correction only and for attenuation correction and fusion images.

## Conclusion

As described above, optimization is promoted in the clinical setting under the newly revised medical law enforcement regulations. The Government, J-RIME, and its liaison organizations need to work together to promote understanding of the DRLs to avoid a misunderstanding that the objective of DRLs is dose reduction. This is because the concept of medical radiation protection has not yet fully penetrated in Japan.

For optimization of protection using DRLs, the dose at one's own institution is required to be compared with the DRLs. However, it is difficult if no dosimeter is available. The provision of technical support to clinical settings, such as a rental service for dosimeters and phantoms, may be needed.

The next step is to develop an infrastructure for the review of DRLs at certain intervals and to offer advice regarding incorporation into domestic regulation systems.
